# Perspectives of Orthoptists Working with Patients with Communication Impairments

**DOI:** 10.22599/bioj.321

**Published:** 2024-01-03

**Authors:** Sonia Lau, Emma Power, Amanda French

**Affiliations:** 1 Discipline of Orthoptics, Graduate School of Health, University of Technology Sydney, Sydney, Australia; 2 Discipline of Speech Pathology, Graduate School of Health, University of Technology Sydney, Sydney, Australia

**Keywords:** vision impairment, communication impairment, supportive communication, patient-centred care, education

## Abstract

**Aims:**

To survey orthoptists’ confidence in communicating with patients with communication impairments and to investigate resources orthoptists are currently using to aid assessment and management and to explore future resources that may be beneficial.

**Methods and Procedures:**

Practicing orthoptists (n = 63; median age range: 31–35 years old) completed an online survey with quantitative and qualitative questions which investigated approaches to adult and paediatric patients with communication impairments and any communication tools used. Analysis of quantitative survey responses was conducted using IBM SPSS v27. Content analysis of qualitative responses was done.

**Outcomes and Results:**

Simple communication strategies (e.g., eye contact and body language, repeating/rephrasing sentences) were commonly used with both adult and paediatric patients while more complex strategies (e.g., electronic visual aids, writing key words/concepts) were rarely used. Usage of communication strategies was not affected by length of work experience, workplace clinical speciality or training during their clinical degree or after graduation (p < 0.05). Most participants (71.2%) reported being unaware of resources available for orthoptists to assist in the assessment and management of patients with communication impairments.

**Conclusions and Implications:**

Orthoptists have adopted some communication strategies to improve their interactions with patients with communication impairments, despite limited resources. With proper resources, such as training in supportive communication techniques, they can provide optimal patient care, making it essential to identify what kind of resources would be most appropriate.

## Introduction

Visual impairments can occur alongside communication impairments at any age. For example, around 2.5% to 5.3% of children have strabismus (Graham 1974; Macfarlane et al. 1987; Robaei et al. 2006), and 1.9% to 2.5% of children have amblyopia (Friedman et al. 2009; Multi-Ethnic Pediatric Eye Disease Study Group 2008; Pai et al. 2012). Age-related ocular conditions such as cataracts, glaucoma, and age-related macular degeneration occur frequently. Cataracts are the most common, with 68.4% of adults aged 65 to 74 having some type of cataract (Mitchell et al. 1997). This prevalence increases to 90.5% in adults aged 75 to 84 and 98.1% in adults 85 and older (Mitchell et al. 1997). On the other hand, communication disability affects approximately 5.0% of Australians, with 64.8% of them having a mild or moderate communication disability, while the remaining 35.2% have a severe communication disability (Australian Bureau of Statistics 2015). The severity varies with age, with children being more likely to have severe communication disabilities, while adults aged 65 and older are more likely to have mild or moderate communication disabilities (Australian Bureau of Statistics 2015). Among children who have communication disabilities, 86.5% of cases are considered severe (Australian Bureau of Statistics 2015). These range from stammering and speech-sound disorders (McKinnon et al. 2007) to communication disorders associated with intellectual disabilities and autism spectrum disorders (Pinborough-Zimmerman et al. 2007). In adults, 77.5% of those aged 65 and older with communication disability have a mild or moderate communication disability (Australian Bureau of Statistics 2015). These disabilities include aphasia post-stroke, age-related hearing loss and cognitive-communication impairments (Hewetson et al. 2018; Laforge et al. 1992; Pedersen et al. 2004). Individuals may also have both visual and communication impairments concurrently; in the case of stroke, individuals might have post-stroke aphasia together with visual field loss (Pedersen et al. 2004; Rowe 2017).

Orthoptists are eye care allied health professionals who work in private orthoptic and ophthalmology clinics, hospital eye clinics and community and rehabilitation settings. For practicing orthoptists, the most common patient age groups seen in clinic are children and adults aged 65 and older, due to a higher frequency of eye conditions in these patient age groups. As a result, orthoptists may often encounter paediatric and adult patients with specific communication needs.

Few communication aids are available for allied health professionals to assist in the management of patients with communication disorders and none have been evaluated for use by orthoptists. However, there have been notable efforts to expand communication partner training programmes, including Supported Conversation for Adults with Aphasia (SCA™), a face-to-face training programme developed by the Aphasia Institute in Canada (Kagan et al. 2001). Similarly, E-learning Communication Partner Training that has a face-to-face training component, as well as an online component, developed by researchers in Australia (Heard et al. 2017). There are also augmentative and alternative communication (AAC) strategies which increase the communicative accessibility of the healthcare service being accessed by the patient (Hemsley et al. 2014).

Previous studies indicate that communication partner training is beneficial as it increases the confidence of health professionals who interact with people with complex communication needs (Cameron et al. 2015; Cameron et al. 2017; Heard et al. 2017). Furthermore, health professionals with a deeper understanding of different communication strategies and access to communication aids can hold effective conversations with patients and deliver better healthcare services (Heard et al. 2017; Hemsley et al. 2013; Hemsley et al., 2014). Healthcare professionals not provided with this type of training or any other communication aids are likely to find it extremely difficult to communicate with patients with complex communication needs (Hemsley et al. 2001). Patients may also experience difficulty describing their symptoms, thus creating another barrier to accessing optimal healthcare (Hemsley et al. 2001).

Additionally, studies on existing communication training programmes do not include vision professionals. It is unclear if existing programmes are beneficial in assisting healthcare professionals interacting with individuals with dual visual and communication impairments. Communication is extremely important for patient compliance with treatment; when a patient understands how the management plan will help with their condition, they are more inclined to follow it (Taylor et al. 2002). Furthermore, if orthoptists are able to communicate with the patient in a way that involves and supports the patient, trust and rapport is built (Pinto et al. 2012). This ultimately increases the effectiveness of the healthcare service as well as patient compliance (Leach 2005). Therefore, it is valuable to understand orthoptists’ perspectives on working with individuals with dual visual and communication impairments and to determine if further resources or training is required.

This study aims to investigate the current confidence of orthoptists when assessing patients with communication impairments. We aim to determine the resources orthoptists are currently using to aid communication, in addition to future resources they would like to have available.

## Methods

### Participants

The survey was distributed to orthoptists via Orthoptics Australia and the British and Irish Orthoptic Society. Attendees of relevant orthoptic events were also invited to participate. The survey link was distributed with an invitation to participate electronically. Eligibility for the study included qualified orthoptists currently practicing in Australia, the United Kingdom, the United States of America, Canada, or New Zealand.

### Ethics Approval and Informed Consent

Participants indicated informed consent by completing the survey. The survey was completed anonymously, and participants were not required to provide any identifying details. Ethics approval for this study was obtained from the University of Technology Sydney Human Research Ethics Committee (Approval number: ETH21–6667), and the study abides by the tenets of the Declaration of Helsinki.

### Procedures

A review of the literature did not reveal any previously used survey instruments in this area. Thus, an electronic questionnaire was developed based on the researchers experience with orthoptic practice, potential patient groups accessing orthoptic services, communication impairments and strategies to support communication (Appendix A). Additionally, as per online survey guidelines, the survey was piloted prior to being distributed widely (Andrews et al. 2007). Eleven orthoptists affiliated with the University of Technology Sydney Orthoptics faculty were invited to participate in the survey. They were also asked additional questions assessing the feasibility of the survey for potential refinement prior to wider distribution within the orthoptic profession. Feedback from the pilot were used to amend the survey prior to distribution (Appendix B).

The questionnaire consisted of five sections; the first section addressed participant demographics and consisted of 11 primary questions, with four follow up questions based on participant responses. The responses collected information on the participants’ location, age, gender, years practicing as an orthoptist, the type of clinic they worked at, languages spoken and used in practice, and their general confidence in communicating with patients.

The second section contained six questions regarding the participants’ experiences with adult patients. Adult patients were defined as patients who were >18 years of age. Participants were asked to rate the frequency of encountering patients with certain conditions (e.g., stroke, dementia) and communication disorders (e.g., aphasia, age-related communication impairments) on a five-point Likert scale which ranged from never to very often. These conditions and communication disorders were selected as they are the most commonly reported conditions in this age group (Australian Bureau of Statistics 2015; Hewetson et al. 2018; Laforge et al. 1992; Pedersen et al. 2004). Following this, participants were asked to rate their confidence in assessing and managing patients with these specific communication disorders on a scale of one to ten, with one being not confident at all and ten being extremely confident.

Similarly, the third section contained five questions regarding the participants’ experiences with paediatric patients. In this survey, paediatric patients were defined as patients who were <18 years of age. The questions asked participants to rate the frequency of seeing patients with certain communication conditions (e.g., stammering, communication impairments associated with developmental disorders or delays) on the same Likert scale as used for adult patients, ranging from never to very often. As with the adult conditions, these were selected as they were the most common communication impairments in paediatric patients (Australian Bureau of Statistics 2015; McKinnon et al. 2007; Pinborough-Zimmerman et al. 2007). In the survey, the term ‘stutter’ was used as it is the preferred term in Australia. For this paper, the term ‘stammer’ has been used in its place. Participants were again asked to rate their confidence in assessing and managing patients with those communication disorders on a scale of one to ten, with one being not confident at all and ten being extremely confident.

The fourth section consisted of ten questions and eight follow up questions investigating the participants’ methods of interacting with patients who have communication disorders. Additionally, participants were asked whether resources are available to assist in the assessment and management of these patients. Participants had to first rate the frequency in which they used communication strategies to communicate with adult and paediatric patients with communication impairments on a Likert scale ranging from never to very often. These communication strategies were obtained from Morris et al.’s study on patient-centred communication strategies (Morris et al. 2015). In that study, patients with aphasia and their companions were interviewed on behaviours their physicians demonstrated that facilitated effective communication (Morris et al. 2015). Additional strategies not included in Morris et al.’s study, such as the use of electronic visual aids, were added after discussion with the research team. The strategies included in the questionnaire were: speaking slowly, using eye contact and body language, repeating or rephrasing what had been said, using hand gestures, using visual aids (e.g., communication boards), using electronic visual aids (e.g., tablets), writing down key words/concepts, providing time for the patient to communicate, using the patient’s companion/carer as required, using close ended questions, repeating back what was heard to check understanding, using analogies, and writing down instructions.

Participants were then asked about training they have received regarding effective communication with patients with communication impairments, during their clinical degrees and/or after graduation. They were required to rate statements about training on a Likert scale with options from strongly disagree to strongly agree. Using the same scale, participants also rated statements about their awareness of resources available for orthoptists to assist in communicating with patients with communication impairments, and whether they thought that there were sufficient resources. These resources could include existing programmes or bespoke resources available within their workplace. Depending on their response, follow-up questions addressed the type of training, whether it was effective, the types of resources they were aware of, whether they used those resources or why they did not think there were sufficient resources.

Additionally, participants were asked to rate the difficulties they experience when assessing and managing patients with communication impairments. This information was collected on a scale of one to ten, with one being no difficulty and ten being extreme difficulty. They had to rate different aspects of patient-clinician interactions (e.g., establishing rapport, explaining the findings and implications of their assessment). The last three questions of this section were open ended questions enquiring about the participants’ experiences with patients with communication impairments and resources.

The final section contained three optional questions which surveyed interest in further research. The first question surveyed participant interest in various types of training options for orthoptists to learn more about accessible communication. The second question allowed general feedback while the last question offered interested participants the opportunity to participate in further research.

### Statistical Analysis

Data collected from the second round of distribution was extracted and compiled in Microsoft Excel. Analysis of quantitative survey responses was conducted using IBM SPSS v27. Analysis of Variance (ANOVA) was used to compare the different variables (for example, participant confidence across categories of work experience in years). Percentages were calculated for data that could not be analysed and presented descriptively. Content analysis of qualitative responses was created by the second author and discussed with the third author to create the final categories (Hsieh & Shannon 2005). Design and reporting of the study were guided by the CHEcklist for Reporting Results of Internet E-Surveys (CHERRIES) (Eysenbach 2004). See Appendix C for the CHERRIES.

## Results

### Participant Demographics

**Figure 1:**
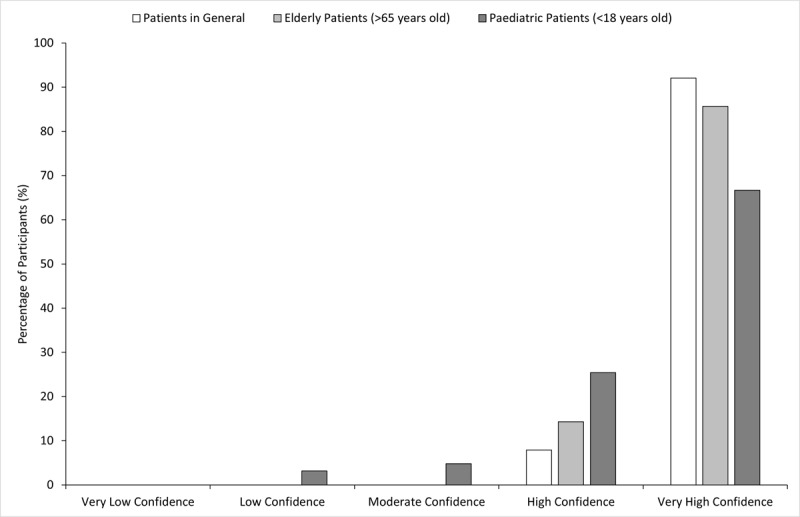
**Participants’ rating of their confidence in communicating with different patient groups**. Participants (n = 63) were asked to rate their confidence in communicating with patients in general, elderly patients (>65 years old) and paediatric patients (<18 years old) on a scale of 1 to 10. It was then classified into very low confidence (1–2), low confidence (3–4), moderate confidence (5–6), high confidence (7–8) and very high confidence (9–10).

A total of 63 participants responded to the survey. 22.2% of the orthoptists surveyed were ≤25 years old (median age bracket: 31–35 years old) and 84.1% were female. In their career as an orthoptist, 38.1% of participants indicated that they had worked as an orthoptist for ≤5 years and 27.0% indicated that they had worked as an orthoptist for ≥21 years. The most common clinic specialities that participants worked in were general ophthalmology (62.9%), paediatrics (50.0%) and neuro-ophthalmology (48.4%). A little less than half (42.9%) work in a private clinic and 46.0% work in a public hospital (Table 1). Participants generally indicated that they felt confident in communicating with their patients. Figure 1 shows that almost all participants indicated that they had high or very high confidence in communicating with patients in general (100.0%), elderly patients >65 years old (100.0%) and paediatric patients <18 years old (92.1%). Confidence in interacting with general patients and elderly patients was not affected by length of work experience (p > 0.05). However, the more work experience a participant had, the more confident they were in interacting with paediatric patients (p = 0.001).

**Table 1:** Participant Demographics. This table summarises the demographics of participants in this survey (n = 63). Percentages have been rounded to the nearest single decimal place.


	NUMBER OF PARTICIPANTS	PERCENTAGE (%)

**Age**		

≤25 years old	14	22.2

26–30 years old	13	20.6

31–35 years old	11	17.5

36–40 years old	4	6.3

41–45 years old	5	7.9

46–50 years old	5	7.9

≥51 years old	11	17.5

**Gender**		

Female	53	84.1

Male	8	12.7

Non-binary/Gender Diverse	1	1.6

Prefer Not to Say	1	1.6

**Length of Time Working as an Orthoptist**		

≤5 years	24	38.1

6–10 years	10	15.9

11–15 years	7	11.1

16–20 years	5	7.9

≥21 years	17	27.0

**Clinic Speciality**		

General Ophthalmology	39	62.9

Paediatrics	31	50.0

Retina	28	45.2

Cornea	16	25.8

Refractive	18	29.0

Oculoplastics	19	30.6

Neuro-ophthalmology	30	48.4

Low vision	10	16.1

Other	10	16.1

**Work Environment**		

Private Clinic	27	42.9

Public Hospital	29	46.0

Community Health Centre	3	4.8

Low Vision Provider	3	4.8

Other	1	1.6


### Adult (>18 years old) and Paediatric Patients (<18 years old) with Communication Impairments

Most participants (57.1%) indicated that they worked with adult patients with noticeable communication impairments that impact their clinical assessment and management often or very often, with the most frequent communication impairment being age related. Majority of participants (81.0%) responded that they encounter patients who have age-related communication impairments often or very often (Table 2). Individuals with a stammer were encountered the least frequently, with 61.9% of responses showing that they never or rarely see these patients in clinic (Table 2).

**Table 2:** **Participants’ rating of the frequency in which they encounter adult patients (>18 years old) and paediatric patients (<18 years old) with the following communication impairments**. Participants (n = 63) rated the frequency in which they see patients with these communication impairments in clinic on a scale from never to very often. Categories are ranked according to percentage of participants who rated the frequency of encountering that communication impairment as very often.


	NEVER (%)	RARELY (%)	SOMETIMES (%)	OFTEN (%)	VERY OFTEN (%)

**Adult patients (>18 years old)**					

Age-Related Communication Impairments	4.8	1.6	12.7	28.6	52.4

Combination of Two or More Impairments	4.8	14.3	46.0	25.4	9.5

Aphasia	6.3	23.8	47.6	12.7	9.5

Developmental Disorders or Delays	4.8	23.8	36.5	27.0	7.9

Cognitive-Communication Impairments	7.9	23.8	39.7	20.6	7.9

Dysarthria	3.2	41.3	33.3	17.5	4.8

Stammers	3.2	58.7	27.0	9.5	1.6

Apraxia	12.7	42.9	36.5	6.3	1.6

**Paediatric patients (<18 years old)**					

Autism Spectrum Disorder	5.0	15.0	21.7	33.3	25.0

Language Disorders	10.0	20.0	30.0	16.7	23.3

Developmental Disorders or Delays	13.3	16.7	25.0	25.0	20.0

Reduced Literary Skills	13.3	15.0	28.3	23.3	20.0

Communication Impairments Associated with Chromosomal Disorders	8.3	15.0	26.7	31.7	18.3

Combination of Two or More Impairments	8.3	18.3	31.7	23.3	18.3

Reduced Proficiency in English	8.3	28.3	23.3	21.7	18.3

Social Communication Disorder	8.3	21.7	30.0	23.3	16.7

Speech Disorders	11.7	25.0	36.7	10.0	16.7

Stammers	13.3	45.0	28.3	3.3	10.0

Hearing Impairments	11.7	28.3	36.7	18.3	5.0


**Figure 2:**
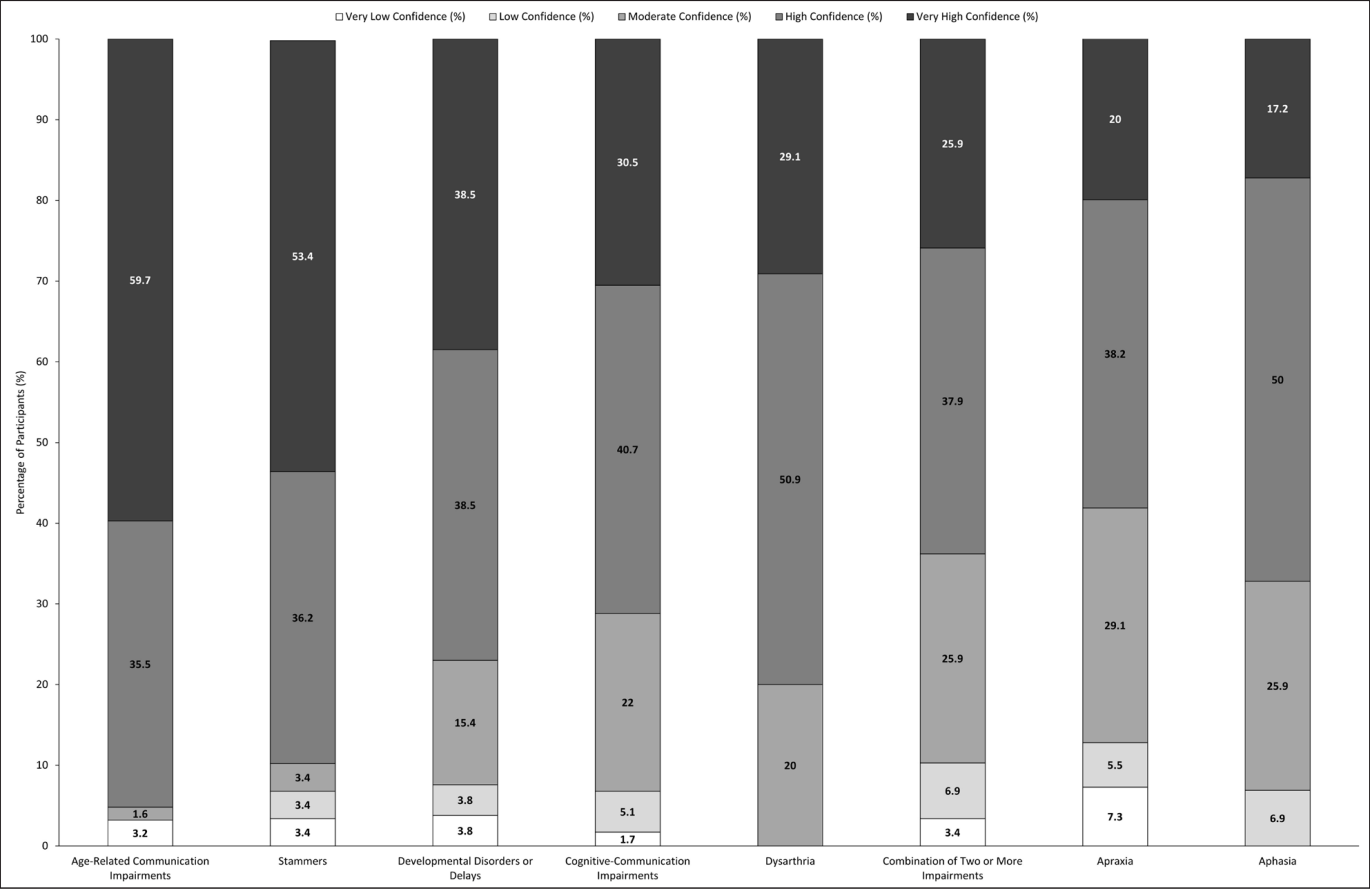
**Participants’ rating of their confidence in communicating with adult patients (>18 years old) with various communication impairments**. Participants (n = 63) rated their confidence in communicating with patients with communication impairments on a scale of 1 to 10. Responses were classified as very low confidence (1–2), low confidence (3–4), moderate confidence (5–6), high confidence (7–8) and very high confidence (9–10). For participants who indicated that they had never seen patients with that particular communication impairment, a non-applicable option was available. Categories are ranked according to percentage of participants who rated their confidence in communicating with patients with that communication impairment as very high.

Overall, at least 58.2% of respondents reported high or very high confidence for all eight communication impairments (Figure 2). Participants were the most confident in interacting with individuals with age-related communication impairments, 95.2% of responses indicated that they had high or very high confidence (Figure 2). Less than 10.0% reported low or very low confidence across communication impairments, except for individuals with apraxia (12.8%) and individuals who have a combination of communication impairments (10.3%) (Figure 2).

In the paediatric patient group, 45.9% of participants encounter paediatric patients with noticeable communication impairments that impact their clinical assessment often or very often. The most common communication impairment was associated with autism spectrum disorder, with 58.3% of participants encountering these patients often or very often (Table 2). Like in the adult population, paediatric patients with a stammer were the least frequently seen by participants in clinic; 58.3% of responses indicated that they never or rarely see these patients in clinical practice (Table 2).

**Figure 3:**
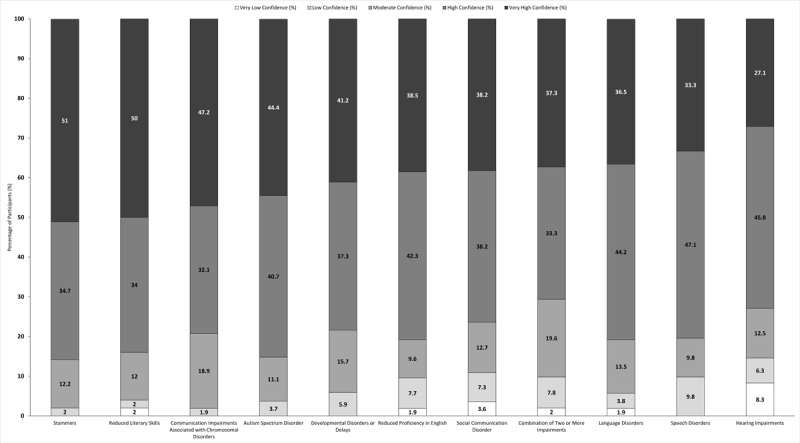
**Participants’ rating of their confidence in communicating with paediatric patients (<18 years old) with various communication impairments**. Participants (n = 18) rated their confidence in communicating with patients with communication impairments on a scale of 1 to 10. Responses were classified as very low confidence (1–2), low confidence (3–4), moderate confidence (5–6), high confidence (7–8) and very high confidence (9–10). For participants who indicated that they had never seen patients with that particular communication impairment, a non-applicable option was available. Categories are ranked according to percentage of participants who rated their confidence in communicating with patients with that communication impairment as very high.

Participants reported being more confident in interacting with paediatric patients with communication impairments, more than 70.6% of participants had high or very high confidence in communicating with patients with any of the eleven communication impairments (Figure 3). Less than 10.0% of participants reported low or very low confidence in communicating with these populations, except for individuals with social communication disorder (10.9%) and individuals with a hearing impairment (14.6%) (Figure 3).

Confidence in interacting with adult patients with these communication impairments was not affected by length of work experience (p > 0.05). Confidence was affected by length of work experience for paediatric patients with autism spectrum disorder (p = 0.043), developmental disorders or delays (p = 0.049), communication impairments associated with chromosomal disorders (p = 0.006) and reduced proficiency in English (p = 0.021). For these populations, as length of work experience increased, confidence in communicating with these patients increased. Confidence was also not affected by training before or after graduation (p > 0.05).

However, workplace clinical speciality significantly impacted confidence, with participants who worked in a clinic that specialised in paediatrics having higher confidence in communicating with paediatric patients with social communication disorder (p = 0.009). Participants who worked in neuro-ophthalmology clinics had higher confidence in communicating with adults with aphasia (p = 0.048) and dysarthria (p = 0.029), and in children, communication impairments associated with developmental disorders and delays (p = 0.011) or chromosomal disorders (p = 0.024).

### Communication Strategies

In adults, the most commonly used communication strategies were eye contact and body language, and providing time for the patient to communicate. These communication strategies were used very often by 69.5% and 64.4% of participants, respectively (Table 3). The least common communication strategies were electronic visual aids and writing down key words/concepts, with 52.5% of participants never or rarely using electronic visual aids and 37.3% never or rarely writing down key words/concepts (Table 3).

**Table 3:** **Participants’ rating of the frequency in which they use various communication strategies with adult patients (>18 years old)**. Participants (n = 63) rated the frequency in which they use communication strategies with adult patients from never to very often. Categories are ranked according to percentage of participants who rated the frequency of using that communication strategy as very often.


	NEVER (%)	RARELY (%)	SOMETIMES (%)	OFTEN (%)	VERY OFTEN (%)

Eye Contact and Body Language	3.4	0.0	5.1	22.0	69.5

Providing Time for Patient to Communicate	3.4	1.7	3.4	27.1	64.4

Repeating/Rephrasing What was Said	1.7	0.0	3.3	38.3	56.7

Using Patient’s Companion/Carer	3.4	0.0	0.0	42.4	54.2

Close-Ended Questions	3.4	1.7	5.1	39.0	50.8

Hand Gestures	3.4	3.4	1.7	42.4	49.2

Speaking Slowly	3.3	0.0	11.7	40.0	45.0

Repeating to Check Understanding	3.4	0.0	8.5	50.8	37.3

Analogies	3.4	8.5	32.2	27.1	28.8

Visual Aids	5.1	10.2	32.2	27.1	25.4

Writing Down Instructions	6.8	16.9	30.5	30.5	15.3

Electronic Visual Aids	20.3	32.2	27.1	11.9	8.5

Using Key Words	11.9	25.4	42.4	13.6	6.8


The communication strategy that was used most often with paediatric patients was using the patient’s companion as necessary, and eye contact and body language; 67.2% of participants use the patient’s companion very often and 58.6% use eye contact and body language very often (Table 4). Similar to adult patients, writing down key words/concepts and using electronic visual aids were the least used communication strategies, with 65.5% and 58.6% respectively never or rarely using these strategies (Table 4).

**Table 4:** **Participants’ rating of the frequency in which they use various communication strategies with paediatric patients (<18 years old)**. Participants (n = 63) rated the frequency in which they use communication strategies with paediatric patients from never to very often. Categories are ranked according to percentage of participants who rated the frequency of using that communication strategy as very often.


	NEVER (%)	RARELY (%)	SOMETIMES (%)	OFTEN (%)	VERY OFTEN (%)

Using Patient’s Companion/Carer	5.2	1.7	6.9	19.0	67.2

Eye Contact and Body Language	5.2	8.6	5.2	22.4	58.6

Providing Time for Patient to Communicate	5.2	6.9	3.4	37.9	46.6

Close-Ended Questions	5.2	5.2	8.6	34.5	46.6

Hand Gestures	6.9	6.9	6.9	32.8	46.6

Visual Aids	6.9	10.3	8.6	29.3	44.8

Repeating/Rephrasing What was Said	5.2	10.3	8.6	34.5	41.4

Repeating to Check Understanding	5.2	3.4	20.7	34.5	36.2

Speaking Slowly	5.2	13.8	15.5	34.5	31.0

Analogies	10.5	8.8	24.6	29.8	26.3

Electronic Visual Aids	24.1	34.5	17.2	6.9	17.2

Writing Down Instructions	15.8	40.4	19.3	14.0	10.5

Using Key Words	20.7	44.8	22.4	6.9	5.2


Usage of these communication strategies was not affected by length of work experience, workplace clinical speciality, training during their clinical degree or after graduation for adult and paediatric patients (p > 0.05).

### Difficulties Experienced

**Figure 4:**
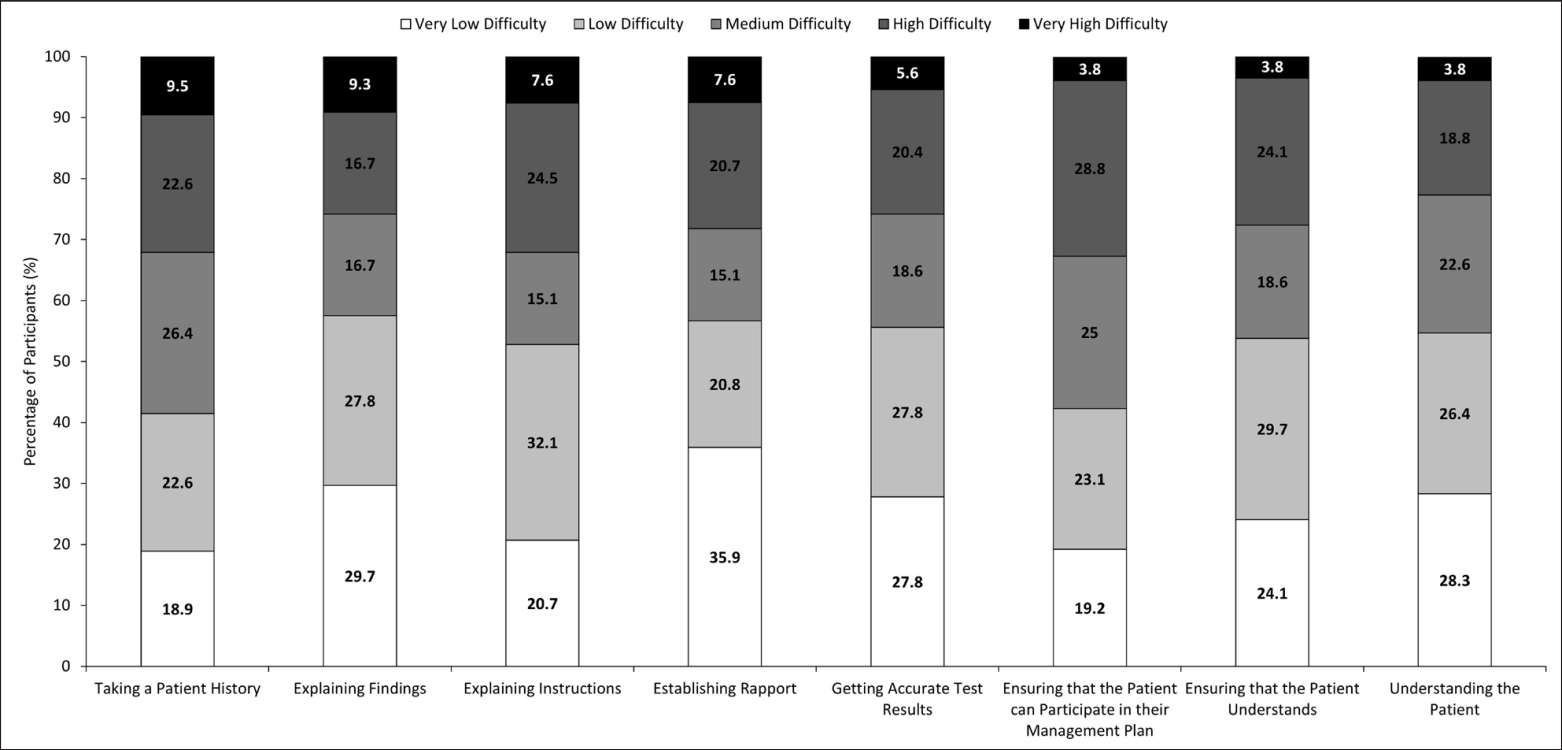
**Participants’ rating of the difficulties they experience when they are assessing and managing patients who have communication impairments**. Participants (n = 63) were asked to rate the difficulties they experience with patients who have communication impairments on a scale of 1 to 10. It was then classified into very low difficulty (1–2), low difficulty (3–4), moderate difficulty (5–6), high difficulty (7–8) and very high difficulty (9–10). Categories are ranked according to percentage of participants who rated difficulty of that task as high difficulty.

Participants generally did not report experiencing marked difficulty in any aspect of their clinical role. Of the participants, 9.5% rated taking a patient history as a very high difficulty task, while 3.8% rated ensuring that the patient can participate in their management plan, ensuring that the patient understands what is being said and understanding the patient as being very high difficulty (Figure 4). However, about half of all participants experienced some sort of difficulty with all these tasks (Figure 4). Difficulties experienced was not affected by length of work experience, workplace clinical speciality or training during their clinical degree or after graduation (p > 0.05).

### Training and Resources

Most participants (57.6%) indicated that they received some training in communicating with patients with communication impairments during their clinical degree. This was mainly through clinical placements, where they had the opportunity to interact with patients with communication impairments under the supervision of a qualified orthoptist. However, 52.6% had not completed additional training in this area after graduating from their clinical degrees. All participants who indicated they had received training after graduation mentioned that it was on the job training where they consulted their colleagues to learn how to best communicate with these patients.

Majority of the participants (71.2%) reported that they are unaware of any resources available for orthoptists to aid in assessment and management of patients with communication impairments. However, 69.7% of participants felt that there were sufficient resources available.

### Qualitative Responses

Qualitative questions in the survey focused on a few areas: communication impairments encountered in clinical practice, communication strategies, training and resources, and challenges experienced (Table 5).

**Table 5:** **Qualitative Responses**. This table summarises the main concepts in participants’ responses to the qualitative questions and notes the frequency of the idea mentioned (n = 63).


CONCEPT	NUMBER OF TIMES MENTIONED

**Q8a: As you do not have access to interpreters for patients who cannot speak English, please explain how you complete the session with the patient in the absence of an interpreter**.	

Hand gestures and body language	4

Using the patient’s family member/friend	3

Google Translate	3

Simplifying words used	2

Non-verbal response strategies (e.g. matching cards)	1

**Q16a: Which communication impairments are they? (Additional communication impairments encountered in adult patients which were not included in the list)**	

Hearing impairment (includes deafness, lip-reading)	8

Mutism/Selective-mutism	3

Autism (non-verbal)	2

Communication impairment associated with developmental delays	1

**Q25a: What kind of training was it? (Training that they have received)**	

Colleagues (other eyecare professionals)	12

Workplace training modules/Training from other healthcare professionals	11

Clinical placements	5

On-the-job practical experiences	4

Online courses	2

**Q25c: Why not? (Training was not effective)**	

Limited time and practical experiences	4

No exposure to resources	2

Clinical placement did not allow interactions with highly complex patients	1

**Q26a: Please identify these resources. (Resources that they have access to)**	

Visual aids (e.g. communication boards, picture boards, leaflets)	14

Interpreters	6

Non-verbal supports (e.g. AUSLAN, Makaton)	6

Other healthcare professionals	5

Internet, online courses, and apps	4

Modified assessment methods	3

Training	2

Patient’s companion or carer	2

**Q27a: Why not? (Resources are not sufficient)**	

Don’t know where to find resources	8

Difficulty accessing resources	7

Absence of orthoptic specific resources	3

**Q30: Can you provide an example of a time where you utilised communication strategies or supported communication to effectively communicate and enhance patient care? (Supported communication is communication that uses techniques to encourage conversation with patients with communication difficulties through spoken and written keywords, body language and gestures, drawings and pictographs)**	

Non-verbal supports (e.g. hand gestures, body language, sign language, Makaton)	13

Written supports (e.g. templates for questions and possible answers, Tumbling E chart, communication via writing, leaflets)	10

Communication aids (e.g. language boards)	8

Pictoral supports (e.g. drawing images/pictures)	7

Language translation supports (e.g. interpreters, Google Translate)	5

Changes to verbal communication (e.g. close ended questions, altered speech)	2

Joint assessment with speech pathologist	1

**Q31: Can you provide an example of a time where communication with a patient was difficult and describe the impact it had on your assessment or management of the patient?**	

Communication impairments	

Dementia	6

Hearing impairment (including deafness, hard-of-hearing)	7

Autism	5

Aphasia	4

Communication impairment associated with Down syndrome	2

Communication impairment associated with cerebral palsy	1

Communication impairment associated with developmental delay	1

Communication impairment associated with acquired brain injury	1

Parkinson’s	1

Difficulties	

Engaging patient	6

Using objective tests instead	4

Increased time needed for assessments – breaks, communicating with carer	4

Explaining instructions	4

Gauging accuracy of history taken	3

Patient fatigue/frustration	3

Understanding patient	2

**Q32: What types of resources would you like to have in the future to make communicating with these patients easier?**	

Training in communication strategies (speech pathologist approved), knowledge of common conditions	7

Electronic devices (e.g. iPads) and apps	6

Access to translators	3

Communication aids	2

Ability to seek information from other healthcare professionals	2

Interactive resources (e.g. visual stimulation added to current visual acuity testing)	1

Resources to further engage paediatric patients with communication impairments)	1

Lectures at conferences	1


Most participants mentioned using communication strategies that were aimed at helping the patient understand what they were saying rather than helping the patient respond. These strategies involved modifying the way information was presented to the patient. The most common modification was non-verbal supports which were mentioned 13 times. Some examples given by participants are ‘commonly use sign language learnt many years ago’ and ‘I frequently use hand gestures to explain strabismus to parents and the concept of fixation’. The next most common modification was written supports. One participant gave an example where ‘I have a patient who was born with no ears. I have set in place templates to provide both questions and some possible answers and explaining each test to assist during the patient consultation’. This was followed by communication aids such as matching boards to aid in their assessment of the patient.

Participants were also asked to provide examples of a challenging experience they have had involving patients with communication impairments. Engaging the patient was the most common challenge, followed by explaining instructions, increased time needed for the assessment and the need to use objective tests for the assessment. For example, ‘Assessing a hard of hearing patient’s vision. Without a sign interpreter present, I struggled to build rapport with the patient. In order to assess the patient’s vision, he had to point to a piece of paper and pen so he could write the letters down’ while another participant mentioned ‘Subjective refraction, patient with Down syndrome – difficult to understand patient’s sounds. Ideally patient needed updated prescription, time frame and fatigue were becoming a problem’. These challenges can impact the clinical management of a patient. Another participant gave an example where an ‘Older patient with dementia who was also hard of hearing. Did not understand my instructions and patient was becoming confused with why/where they were. This led to being unable to get imaging of the eye and the patient had an acute retinal bleed so diagnosis/management was ultimately impacted’.

Although participants mentioned clinical placements as the main resource for them to learn how to communicate with patients with communication impairments, one participant mentioned that ‘only better performing students are given the opportunity to assess complex patients’ and even then, it would be ‘under the oversight of a supervisor which restricts the student’s capacity to communicate’. Additionally, on clinical placements there is a ‘limited amount of time’ for students to communicate with the patient. Participants who felt that there were not enough resources available ‘did not know where to find [resources] directed at orthoptists’. They also mentioned that there were ‘no orthoptic specific communication books … [They] only have the general communication books … from the stroke team’.

## Discussion

This study is the first to investigate the interaction of orthoptists with patients that have communication impairments and the availability and need for orthoptists to have additional training and resources in this area. We have found that the majority of respondents encounter both adult and paediatric patients with noticeable communication impairments that impact their assessment and management. Unsurprisingly, the results showed that as the frequency in which orthoptists encounter patients with a certain communication impairment increases, their confidence in assessing and managing them increases. This is further supported by previous studies which found that theoretical knowledge about communication impairments is not enough to increase the confidence of allied healthcare professionals (Cameron et al. 2015; Finch et al. 2013). These studies have suggested that practical exposure to patients with communication impairments would be more beneficial in increasing confidence (Finch et al. 2013). One participant in the current study stated that they ‘need more hands on experience to be proficient. I imagine it is hard to provide this’. when questioned about whether the training they have had in communicating with patients with communication impairments was sufficient. These results are consistent with the idea that orthoptists who have had more exposure to patients with complex communication needs tend to have greater confidence in providing the best possible care.

Simple communication strategies to assist in effective communication, such as eye contact and body language, and repeating or rephrasing what has been said, were reported to be often utilised. However, more complex communication strategies like the use of electronic visual aids or writing down key words/concepts were never or rarely used. This finding was consistent between both adult and paediatric patients. This is supported by the responses received when questioned about training and resources; most participants felt that they had not received sufficient training regarding supported communication. Participants felt that there was a lack of resources for orthoptists in this area. This further demonstrates how orthoptists are currently assessing and managing these patients without proper supports, which may mean that this population is not receiving optimal healthcare.

It is important to note that training in this area as well as knowledge of various communication strategies and access to communication aids have been demonstrated to be beneficial for effective communication (Cameron et al. 2017; Cameron et al. 2015; Heard et al. 2017; Hemsley et al. 2014; Hemsley et al. 2013). For patients with complex communication needs, it is essential that they are able to communicate effectively with their healthcare professionals to ensure that their health care needs are adequately met. If they are having difficulty expressing their concerns about their condition, it could potentially result in the patient not receiving optimal care (Hemsley et al. 2001). This can be seen in one participant’s response where they brought up an example of an older patient with dementia who was also hard of hearing who did not understand instructions, leading to the orthoptist being unable to get images of the patient’s eye. The patient had an acute retinal bleed and without the images, their management was affected. Additionally, this could lead to the patient experiencing feelings of frustration and helplessness as they feel like they are unable to understand their healthcare professional or be understood by them (Fried-Oken et al. 1991).

Despite the general absence of training, most participants were utilising some form of communication strategy with these patients. One example is utilising the patient’s companion as necessary (Morris et al. 2015), which this respondent did: ‘Teaching convergence exercises to a stroke patient was tricky. Had to involve her husband in the management to aid with her pen to nose and spot card’. Other strategies include gestures, matching boards and close-ended questions (Morris et al. 2015). For example, one participant mentioned the use of a ‘matching board which worked well with [a patient’s] aphasia’. Another participant stated, ‘I often speak slowly and loudly to patients with hearing problems. To reinforce directions, I also use hand gestures to support my instructions’.

Besides these general communication strategies, there were also alternate strategies being utilised, such as the use of ‘joint assessments with a speech and language therapist’ in order to better communicate with the patient. One participant has even ‘developed a vision passport to give to stroke patient that has pictures and wording agreed with a speech and language therapist’. For patients who are of a non-English speaking background, some participants have found that using applications such as Google Translate can assist in ensuring that the patients understand the instructions they are given.

As participants were the least confident in communicating with patients with post-stroke aphasia in adults and paediatric patients with hearing impairments, the literature was reviewed for any currently available resources for these patient populations. For adults with post-stroke aphasia, there are face-to-face communication partner training programmes like the Supported Conversation for Adults with Aphasia (SCA™) programme (Kagan et al. 2001). For paediatric patients with hearing impairments, there are sign language courses available. Unfortunately, these are not freely available, and these resources do not take into consideration dual visual and communication impairments. Further research should consider resources that are more inclusive and more accessible to healthcare professionals.

Being a preliminary study, future research into this area should expand on these results. The sample size for this study was small and it only included orthoptists who practiced in Australia and the United Kingdom, but as it is a new area of research, the results obtained from this study add interesting findings to the literature. In addition, it would be interesting to investigate whether the orthoptist’s perception of the patient-healthcare professional interaction is the same as the patient’s. Despite most respondents indicating that they have not had training, they do not generally feel that they have experienced much difficulty in interacting with these patients. It would be eye-opening to find out whether the strategies being used by orthoptists are beneficial to the patient. Gaining a better understanding of the patient’s perspective would improve the quality of the healthcare being provided to them.

Additionally, investigating the perspectives of speech pathologists would be beneficial as well. It would be interesting to see how they interact with patients with dual visual and communication impairments, and whether they have found or developed effective ways to communicate with this population. This could possibly encourage interdisciplinary interactions which would ideally optimise these patients’ healthcare.

In conclusion, although the orthoptists who participated in this survey have had limited training, they have adapted some communication strategies to better facilitate communication with patients who have communication impairments. However, with further training in communication, the care being provided to these patients could potentially be improved. Being able to provide the best possible care for patients is an essential part of being a healthcare professional, but care is also an evolving idea that requires more resources as it develops. It is therefore crucial to identify the areas in which health professionals require resources in order to keep providing optimal healthcare to patients.

## Data Accessibility Statement

We do not intend to publicly share the data as it is not allowable based on our ethics approval. Even though it is anonymous data, given that this is a small profession, there is some potential for individual participants to be identified as we have information on demographics, clinical practice, and confidence. If identified, this data may be sensitive.

## Additional File

The additional file for this article can be found as follows:

10.22599/bioj.321.s1 Supplementary File 1. Appendix A-C.
