# Supplementary material for: Perspectives of Orthoptists Working with Patients with Communication Impairments
Source: Br Ir Orthopt J. 2024 Jan 3;20(1):16–30. doi: 10.22599/bioj.321 (PMC10768565; doi:10.22599/bioj.321)
Supplement: Supplementary File 1. — Appendix A-C. [file bioj-20-1-321-s1.pdf]

Appendix A

Final Version of Survey: Perspectives of Orthoptists Working with Patients with Communication Impairments

1) Participant Demographics

No.	Question	Answer
1	Participant Information Statement	Yes, I consent to participating in this study<br>No, I do not consent [skip to end of survey if selected]
2	Are you an orthoptist that is currently located in Australia?	Yes [skip to Q3 if selected]<br>No
2a	Where are you located?	United Kingdom<br>United States of America<br>Canada<br>New Zealand<br>Other [skip to end of survey if selected]
3	What is your age?	≤ 25 years old<br>26 – 30 years old<br>31 – 35 years old<br>36 – 40 years old<br>41 – 45 years old<br>46 – 50 years old<br>≥ 51 years old
4	With what gender do you identify?<br>*Gender refers to current gender, which may be different to sex recorded at birth and may be different to what is indicated on legal documents.*	Male<br>Female<br>Non-binary/Gender diverse<br>Prefer not to say<br>Other [free text response enabled]
5	How long have you been working as an orthoptist?	≤ 5 years<br>6 – 10 years<br>11 – 15 years<br>16 – 20 years<br>≥ 21 years
6	Do you speak any of the following languages in addition to English?<br>*Choose any that apply.*	No, only English<br>Mandarin<br>Arabic<br>Cantonese<br>Vietnamese<br>Italian<br>Greek

		Other [free text response enabled]
7	Are there any languages other than English you use to communicate with patients as part of your clinical role?<br>*Choose any that apply.*	No, only English<br>Mandarin<br>Arabic<br>Cantonese<br>Vietnamese<br>Italian<br>Greek<br>Other [free text response enabled]
7a	[Display this question if 'Mandarin', 'Arabic', 'Cantonese', 'Vietnamese', 'Italian', 'Greek' or 'Other' is selected in Q7]<br>As you do use a language other than English to communicate with patients, what is the context of this?	Free text response
8	Do you have access to interpreters for patients who cannot speak English?	Yes<br>No
8a	[Display this question if 'No' is selected in Q8]<br>As you do not have access to interpreters for patients who cannot speak English, please explain how you complete the session with the patient in the absence of an interpreter.	Free text response
9	What speciality best describes the clinic you primarily work at?<br>*Choose any that apply. If you are currently not working as an orthoptist or are on leave from work, please answer according to your last orthoptic position.*	General ophthalmology<br>Paediatrics<br>Retina<br>Cornea<br>Refractive<br>Oculoplastics<br>Neuro-ophthalmology<br>Low vision<br>Other [free text response enabled]
10	Which of the following best describes the setting in which you primarily work?<br>*If you are not currently working as an orthoptist or are on leave from work, please answer according to your last orthoptic position.*	Private clinic<br>Public hospital<br>Private hospital<br>Rehabilitation center<br>Community health center<br>Low vision provider<br>Other [free text response enabled]
10a	[Display this question if 'Public hospital' or 'Private	Yes

	hospital' was selected in Q10]<br>As you work in a hospital environment, do you ever attend to patients within a ward environment?	No
11	On a scale of 1 to 10, how confident are you in communicating with the following groups of patients to achieve optimal care and outcomes for them?<br>*1 being not confident at all and 10 being extremely confident.*<br>1) Patients in general<br>2) Elderly patients (>65 years of age)<br>3) Paediatrics patients	Scale of 1 – 10

2) Adult patients

No.	Question	Answer
12	How often do you see adult patients (18 years and older) who present with the following conditions?<br>1) Stroke<br>2) Acquired brain injury<br>3) Dementia (e.g. Alzheimer's disease)<br>4) Mild cognitive impairments<br>5) Other degenerative neurological disorders (e.g. motor neuron disease)<br>6) Developmental disorders or delays	Very often<br>Often<br>Sometimes<br>Rarely<br>Never
13	How often do you see adult patients with noticeable communication impairments that impact the way you assess and/or manage them?	Very often<br>Often<br>Sometimes<br>Rarely<br>Never
14	How often do you see patients who present with the following communication difficulties?<br>1) **Aphasia** - impaired language including difficulty with understanding and retrieving words, constructing sentences, reading and writing<br>2) **Dysarthria** - slurred speech<br>3) **Apraxia** - inability to move the face and tongue muscles correctly to form words<br>4) **Cognitive-communication impairments -** impaired interpersonal social communication such as difficulty in turn-taking or verbose/tangential language due to a cognitive deficit<br>5) **Stutters** - speech impairment that may result in the repetition of words or phrases<br>6) **Age-related communication impairments** (e.g. age-related hearing loss)	Very often<br>Often<br>Sometimes<br>Rarely<br>Never

	7) **Developmental disorders or delays** (e.g. cerebral palsy, foetal alcohol syndrome, spina bifida)<br>8) A combination of any two or more of the above conditions	
15	On a scale of 1 to 10, how confident are you in communicating with patients who have each of the following communication difficulties?<br>*1 being not confident at all and 10 being extremely confident.*<br>1) **Aphasia** - impaired language including difficulty with understanding and retrieving words, constructing sentences, reading and writing<br>2) **Dysarthria** - slurred speech<br>3) **Apraxia** - inability to move the face and tongue muscles correctly to form words<br>4) **Cognitive-communication impairments** - impaired interpersonal social communication such as difficulty in turn-taking or verbose/tangential language due to a cognitive deficit<br>5) **Stutters** - speech impairment that may result in the repetition of words or phrases<br>6) **Age-related communication impairments** (e.g. age-related hearing loss)<br>7) **Developmental disorders or delays** (e.g. cerebral palsy, foetal alcohol syndrome, spina bifida)<br>8) A combination of any two or more of the above conditions	Scale of 1 – 10<br>Not applicable option available
16	Are there any other communication impairments you have encountered that was not mentioned?	Yes<br>No
16a	[Display this question if 'Yes' is selected for Q16]<br>Which communication impairments are they?	Free text response
16b	[Display this question if Q16a is displayed]<br>How often do you see patients who present with the communication difficulties you mentioned in Q16a?	Very often<br>Often<br>Sometimes<br>Rarely<br>Never
16c	[Display this question if Q16b is displayed]<br>On a scale of 1 to 10, how confident are you in communicating with patients who have the communication difficulties you mentioned in Q16a?<br>*1 being not confident at all and 10 being extremely confident.*	Scale of 1 – 10
17	Please rate how much you agree or disagree with the	Strongly agree

	following statement:<br>"The clinic at which I work at provides an environment which is accessible for adults with communication impairments through resources such as modified written resources, supported communication and trained staff."	Agree<br>Disagree<br>Strongly disagree

3) Paediatric patients

No.	Question	Answer
18	How often do you see paediatric patients (0 to <18 years old) with noticeable communication impairments that impact the way you assess and/or manage them?	Very often<br>Often<br>Sometimes<br>Rarely<br>Never
19	How often do you see paediatric patients with the following communication difficulties?<br>1) **Language disorders** (e.g. preschool language disorders, learning difficulties, selective mutism which may lead to difficulties in understanding the child or the child understanding your instructions)<br>2) **Speech disorders** (e.g. dysarthia, speech sound disorders, childhood apraxia of speech which leads to difficulties in understanding the child)<br>3) **Stutters** - speech impairment that may result in the repetition of words or phrases<br>4) **Social communication disorder** - a developmental disorder where the child has difficulties using verbal and non-verbal communication appropriately in social settings<br>5) **Autism spectrum disorder** - a developmental disorder where the child has communication difficulties, specific interests and repetitive behaviour<br>6) **Developmental disorders or delays** (e.g. cerebral palsy, foetal alcohol syndrome, spina bifida)<br>7) **Communication impairments associated with chromosomal disorders** (e.g. Down syndrome, Edwards syndrome, Patau syndrome)<br>8) **Hearing impairments** (e.g. children who are hard of hearing or deaf)<br>9) **Reduced proficiency in English** (i.e. children whose first language is not English)	Very often<br>Often<br>Sometimes<br>Rarely<br>Never

	10) **Reduced literary skills** (i.e. children who speak English but have difficulty reading and/or writing)<br>11) A combination of any two or more of the above conditions	
20	On a scale of 1 to 10, how confident are you in communicating with paediatric patients who have each of the following communication difficulties?<br>*1 being not confident at all and 10 being extremely confident.*<br>1) **Language disorders** (e.g. preschool language disorders, learning difficulties, selective mutism which may lead to difficulties in understanding the child or the child understanding your instructions)<br>2) **Speech disorders** (e.g. dysarthia, speech sound disorders, childhood apraxia of speech which leads to difficulties in understanding the child)<br>3) **Stutters** - speech impairment that may result in the repetition of words or phrases<br>4) **Social communication disorder** - a developmental disorder where the child has difficulties using verbal and non-verbal communication appropriately in social settings<br>5) **Autism spectrum disorder** - a developmental disorder where the child has communication difficulties, specific interests and repetitive behaviour<br>6) **Developmental disorders or delays** (e.g. cerebral palsy, foetal alcohol syndrome, spina bifida)<br>7) **Communication impairments associated with chromosomal disorders** (e.g. Down syndrome, Edwards syndrome, Patau syndrome)<br>8) **Hearing impairments** (e.g. children who are hard of hearing or deaf)<br>9) **Reduced proficiency in English** (i.e. children whose first language is not English)<br>10) **Reduced literary skills** (i.e. children who speak English but have difficulty reading and/or writing)<br>A combination of any two or more of the above conditions	Scale of 1 – 10<br>Not applicable option available
21	Are there any other communication impairments you have encountered that was not mentioned?	Yes<br>No
21a	[Display this question if ‘Yes’ is selected for Q21]	Free text response

	Which communication impairments are they?	
21b	[Display this question if Q21a is displayed]<br>How often do you see patients who present with the communication difficulties you mentioned in Q16a?	Very often<br>Often<br>Sometimes<br>Rarely<br>Never
21c	[Display this question if Q21b is displayed]<br>On a scale of 1 to 10, how confident are you in communicating with patients who have the communication difficulties you mentioned in Q16a?<br>*1 being not confident at all and 10 being extremely confident.*	Scale of 1 – 10
22	Please rate how much you agree or disagree with the following statement:<br>"The clinic at which I work at provides an environment which is accessible for children with communication impairments through resources such as modified written resources, supported communication and trained staff."	Strongly agree<br>Agree<br>Disagree<br>Strongly disagree

4) Resources and training

**No.**	**Question**	**Answer**
23	Overall, in your day-to-day clinical practice how frequently do you use the following strategies to communicate with adult patients (>18 years old) with communication impairments?<br>1) Speaking slowly<br>2) Using eye contact and body language that lets the patient know that you're listening<br>3) Repeating or rephrasing what you have said<br>4) Using hand gestures<br>5) Using visual aids such as communication/alphabet/picture boards<br>6) Using electronic visual aids such as smart phones/tablets<br>7) Writing down key words/concepts<br>8) Providing time for the patient to communicate<br>9) Using the patient's companion/carer as needed, i.e. addressing both the patient and their companion<br>10) Using close ended questions, i.e. asking more specific questions to aid understanding or asking yes/no questions<br>11) Repeating back what you understand the patient said to check your understanding	Very often<br>Often<br>Sometimes<br>Rarely<br>Never

	12) Using analogies to explain concepts<br>13) Writing down instructions	
24	Overall, in your day-to-day clinical practice how frequently do you use the following strategies to communicate with paediatric patients (<18 years old) with communication impairments?<br>1) Speaking slowly<br>2) Using eye contact and body language that lets the patient know that you're listening<br>3) Repeating or rephrasing what you have said<br>4) Using hand gestures<br>5) Using visual aids such as communication/alphabet/picture boards<br>6) Using electronic visual aids such as smart phones/tablets<br>7) Writing down key words/concepts<br>8) Providing time for the patient to communicate<br>9) Using the patient's companion/carer as needed, i.e. addressing both the patient and their companion<br>10) Using close ended questions, i.e. asking more specific questions to aid understanding or asking yes/no questions<br>11) Repeating back what you understand the patient said to check your understanding<br>12) Using analogies to explain concepts<br>13) Writing down instructions	Very often<br>Often<br>Sometimes<br>Rarely<br>Never
25	Please rate how much you agree or disagree with the following statements:<br>1) "I have had training on how to communicate with patients with a range of communication difficulties in my clinical degree."<br>2) "I have had training on how to communicate with patients with a range of communication difficulties after graduating from my clinical degree."	Strongly agree<br>Agree<br>Disagree<br>Strongly disagree
25a	[Display this question if 'Strongly agree' or 'Agree' is selected in Q25]<br>What kind of training was it?	Free text response
25b	[Display this question if 25a is displayed]<br>Do you feel that your training was enough to prepare you to communicate effectively with these patients?	Yes<br>No
25c	[Display this question if 'No' is selected in 25b]<br>Why not?	Free text response
26	Please rate your level of agreement or disagreement with the following statement:<br>"I am aware of resources to assist orthoptists in	Strongly agree<br>Agree<br>Disagree

	communicating with patients who have a communication impairment."	Strongly disagree
26a	[Display this question if ‘Strongly agree’ or ‘Agree’ is selected in Q26]<br>Please identify these resources.	Free text response
26b	[Display this question if 26a is displayed]<br>Do you use any of these resources?	Yes<br>No
26c	[Display this question if ‘No’ is selected in 26b]<br>Why not?	Free text response
27	Please rate your level of agreement or disagreement with the following statement:<br>"I feel that I have sufficient resources to assist me as an orthoptist to effectively communicate with patients with communication difficulties."	Strongly agree<br>Agree<br>Disagree<br>Strongly disagree
27a	[Display this question if ‘Strongly disagree’ or ‘Disagree’ is selected in Q27]<br>Why not?	Free text response
28	On a scale of 1 to 10, when assessing and/or managing a patient with a communication impairment, rate the difficulty you have with the following.<br>*1 being no difficulty and 10 being extreme difficulty.*<br>1) Establishing rapport<br>2) Taking a patient history/finding out about current concerns, symptoms etc.<br>3) Explaining instructions to the patient<br>4) Understanding the patient's responses<br>5) Getting accurate test results<br>6) Explaining the findings and implications of your assessment<br>7) Ensuring that the patient understands your instructions/recommendations<br>8) Ensuring the patient can actively participate in the design and implementation of their management plan	Scale of 1 – 10
29	Are there any other difficulties that you experience when communicating with these patients that was not mentioned in the question above?	Yes<br>No
29a	[Display this question if ‘Yes’ is selected in Q29]<br>What are they?	Free text response
30	Can you provide an example of a time where you utilised communication strategies or supported communication to effectively communicate and enhance patient care? (Supported communication is communication that uses techniques to encourage	Free text response

	conversation with patients with communication difficulties through spoken and written keywords, body language and gestures, drawings and pictographs)	
31	Can you provide an example of a time where communication with a patient was difficult and describe the impact it had on your assessment or management of the patient?	Free text response
32	What types of resources would you like to have in the future to make communicating with these patients easier?	Free text response

5) Further research

No.	Question	Answer
33	Would you be interested in any of the following training options to learn more about ensuring accessible communication with patients who have communication difficulties?<br>1) Online webinar<br>2) Face to face workshop<br>3) Reading materials<br>4) Formal course/Micro-credential<br>5) Other	Strongly agree<br>Agree<br>Disagree<br>Strongly disagree
34	Any further comments?	Free text response
35	If you are interested in participating in any further research about this or similar topics, please enter your email address below. By entering your email address, the researchers involved in the project may contact you with an invitation to participate in future research extending directly from this project or based on a similar topic. You will be under no obligation to participate and you may withdraw your agreement to contact you at any time. We will under no circumstances provide your contact email to any third party.	Free text response

Appendix B

Changes to the Survey

Based on the feedback received from the participants, reviewing the results and discussion with the coauthors, the survey was modified in the following ways before it was distributed to a wider population.

1. The question regarding clinic speciality was amended to be a multiple answer question as it was suggested that being able to see the variety of specialities in which participants work in may have implications on the results.
2. Options for adults who have communication impairments associated with developmental disorders or delays was added to the questions.
3. For questions which provided an "Other" option for participants in the event where they have encountered a particular communication impairment that was not an option, it was removed. Instead, a follow up yes or no question was added. This question was amended to "Are there any other communication impairments you have encountered that was not mentioned?" If participants respond with yes, a second follow up question inquiring the type of communication impairment as well as the participant's rating of the frequency in which it is encountered, and their confidence will be asked.
4. If participants indicate that they have never seen patients with noticeable communication impairments that has impacted the way they were assessed and managed, the survey was amended so it did not automatically skip the section.
5. Other communication strategies such as the use of analogies and writing down instructions was added to the question regarding the use of various strategies with adult and paediatric patients.

Appendix C

CHEcklist for Reporting Results of Internet E-Surveys (CHERRIES)

Item Category	Checklist Item	Y/N	Comments
Design	Describe survey design	Y	The target population is practicing orthoptists in Australia, the United Kingdom, the United States of America, Canada, and New Zealand. The pilot of the survey was distributed to a convenience sample.
IRB (Institutional Review Board) Approval and Informed Consent Process	IRB approval	Y	The study has received ethics approval from the University of Technology Sydney Human Research Ethics Committee.
	Informed consent	Y	The first page of the survey was the informed consent form which has information about the length of the time of the survey, data storage, investigators, and purpose of the study. Participants had to consent to participating in this study to proceed to the rest of the survey.
	Data protection	Y	There was an optional question for participants to give their e-mail addresses if they were interested in participating in further research in this area. All data was stored on the password-protected University of Technology Sydney's Qualtrics server and only the research team has access to the data.
Development and Pre-Testing	Development and testing	Y	As described in method section.
Recruitment Process and Description of the Sample Having Access to the Questionnaire	Open survey versus closed survey	Y	Open survey
	Contact mode	Y	Initial contact with potential participants was made via the Internet (e.g. newsletters, social media) and the survey was also advertised at relevant conferences, as described in method section.
	Advertising the survey	Y	

Survey Administration	Web/E-mail	Y	Web
	Context	Y	Organisations for orthoptists, as described in method section.
	Mandatory/Voluntary	Y	Voluntary
	Incentives	Y	No financial incentives were offered.
	Time/Date	Y	01/02/2021 – 05/05/2022
	Randomisation of items or questionnaires	N	The survey questions were not randomised.
	Adaptive questioning	Y	Adaptive questioning was used, depending on the participants' responses to certain questions, follow up questions would be asked.
	Number of items	Y	53 items
	Number of screens (pages)	Y	11 pages
	Completeness check	Y	Request-response feature selected on Qualtrics.<br>Questions on confidence have a "not applicable" option for participants.
	Review step	Y	There is a "back" button available for participants to review and change their answers.
Response Rates	Unique site visitors	N	Collection of IP addresses and cookies were disabled to protect participants' anonymity.
	View rate (Ratio of unique survey visitors/unique site visitors)	N	
	Participation rate (Ratio of unique visitors who agreed to participate/unique first survey page visitors)	N	
	Completion rate (Ratio of users who finished the survey/users who agreed to participate)	Y	63/80 X 100% = 78.75%
Preventing Multiple Entries from the Same Individual	Cookies used	N	Cookies were disabled to protect participants' anonymity. To avoid inclusion of duplicate entries, responses provided in the demographic section were screened to identify duplicates.
	IP check	N	Collection of IP addresses were disabled to protect participants' anonymity.

	Log file analysis	N	Not applicable.
	Registration	N	Not applicable.
Analysis	Handling of incomplete questionnaires	Y	Completed surveys were analysed. Surveys that were not fully completed but did complete more than the demographics section were also analysed. Surveys that only completed the demographics section were not analysed.
	Questionnaires submitted with an atypical timestamp	N	Not applicable.
	Statistical correction	N	Not applicable.
